# Covert infrared image encoding through imprinted plasmonic cavities

**DOI:** 10.1038/s41377-018-0095-9

**Published:** 2018-11-21

**Authors:** Daniel Franklin, Sushrut Modak, Abraham Vázquez-Guardado, Alireza Safaei, Debashis Chanda

**Affiliations:** 1 Department of Physics, University of Central Florida, 4111 Libra Drive, Physical Sciences Bldg. 430, Orlando, FL 32816 USA; 2 NanoScience Technology Center, University of Central Florida, 12424 Research Parkway Suite 400, Orlando, FL 32826 USA; 3 CREOL, The College of Optics and Photonics, University of Central Florida, 4304 Scorpius St., Orlando, FL 32816 USA

## Abstract

Functional surfaces that can control light across the electromagnetic spectrum are highly desirable. Plasmonic nanostructures can assume this role by exhibiting dimension-tunable resonances that span multiple electromagnetic regimes. However, changing these structural parameters often impacts the higher-order resonances and spectral features in lower-wavelength domains. In this study, we discuss a cavity-coupled plasmonic system with resonances that are tunable across the 3–5 or 8–14 μm infrared bands while retaining near-invariant spectral properties in the visible domain. This result is accomplished by regime-dependent resonance mechanisms and their dependence on independent structural parameters. Through the identification and constraint of key parameters, we demonstrate multispectral data encoding, where images, viewable in the infrared spectral domain, appear as uniform areas of color in the visible domain—effectively hiding the information. Fabricated by large area nanoimprint lithography and compatible with flexible surfaces, the proposed system can produce multifunctional coatings for thermal management, camouflage, and anti-counterfeiting.

## Undercover images: Hiding in plasmonic sight

A new device employing the science of ‘plasmonics’ allows control over the interaction of various wavelengths of light with its components, with implications for camouflage and anti-counterfeit applications. The device, designed by Debashis Chanda and colleagues of the University of Central Florida, is formed of a dielectric layer patterned with regularly-spaced nano-sized holes, sandwiched between a reflective metallic mirror and a thin upper gold layer with holes corresponding to the middle layer’s discs. Images are encoded onto the surface in spun coat films of thermoplastic. Changing the diameters and depths of the holes changes how different wavelengths of light react with the materials. The team was able to tune the device’s parameters in a way that made the surface appear as a uniform block of colour unless viewed through an infrared camera over a specific band, when the image can be seen.

## Introduction

Nanostructured plasmonic materials have promised unique control over the electromagnetic spectrum. With the aid of advanced modeling and fabrication techniques, researchers have demonstrated near-arbitrary tailoring of relevant optical field properties, such as amplitude^1^, phase^2–4^, and wave fronts^5,6^—the applications of which are as diverse as light itself. However, few studies have focused on independently controlling these fundamental properties across multiple electromagnetic frequency regimes. These multispectral systems can enable novel surfaces with combined functionalities; for example, reflective multilayer films that selectively absorb and emit infrared light within transparent atmospheric windows for thermal management^7^ or plasmonic filters with tunable resonances for multispectral color imaging^8–12^. Similar concepts can be applied to camouflage and anti-counterfeiting techniques, where encoded images are only viewable within desired spectral regimes. The resonances in these systems are excited electric and magnetic multipole modes^13^, that are highly dependent on the geometries and dimensions of constituent materials via plasmon hybridization^14^ and plasmon–phonon coupling^15^. This attribute provides a convenient and direct path for arbitrarily engineering a surface’s optical characteristics. However, changing the structural parameters to accommodate one spectral regime often influences the higher-order resonances in lower-wavelength ranges—thereby lacking independent control of optical characteristics within individual spectral regimes.

In this study, we demonstrate a plasmonic system with continuously tunable absorption throughout the mid wave (3–5 μm) and long wave (8–12 μm) infrared atmospheric transparency windows while maintaining near-invariant visible properties. This is achieved via a multilayer cavity-coupled nanostructured system, whose spectral response depends on the interactions among plasmonic resonances, diffraction and cavity feedback. In the infrared regime, light absorption is governed by cavity-induced hybrid localized surface plasmons^16^, which occur on an array of gold holes/disks, whereas the visible domain is dominated by diffraction into Fabry-Perot induced propagating surface plasmon polariton (SPP) modes on the top perforated hole array^17^. The resonance modes of each regime depend on a separate (although not exclusive) set of structure parameters, which we define and explore through finite-difference time-domain (FDTD) numerical simulation. By identifying and varying the appropriate dimensional parameters, we experimentally create an infrared ‘color palette’ and realize images that are visible with infrared cameras but are concealed in the visible domain by pixel-to-pixel invariant plasmonic absorption and diffraction. This study demonstrates the use of cavity-induced plasmonics for multispectral engineering and the potential use of these systems in camouflage and anti-counterfeiting technologies.

## Results

### Quasi-3D plasmonic system

**Fig. 1:**
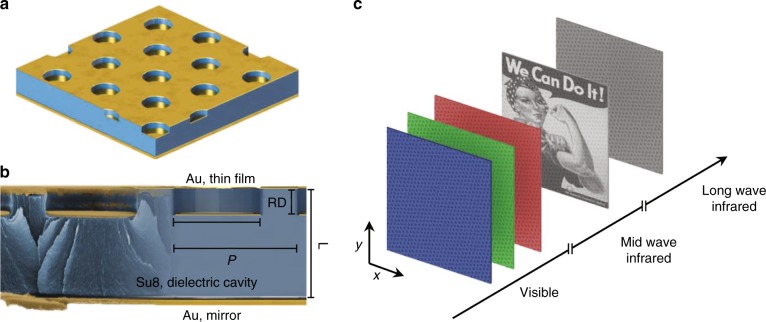
Image encoding and the three-level plasmonic system. **a** Schematic of the cavity-coupled plasmonic device consists of a backmirror, an imprinted array of holes in a polymer, and a second evaporation of gold to create disks and a perforated film. **b** A false-colored scanning electron microscope image of the plasmonic system and a schematic with the various structural parameters. **c** An encoded surface where pixel data are mapped to structural features of the plasmonic system. The spectral axis shows how the data can manifest in the desired wavelength range, as shown in the shortwave infrared window, whereas the surface remains uniform in other windows

A schematic of the cavity-coupled plasmonic system is shown in Fig. 1a. The device consists of a reflective back mirror, a dielectric spacer imprinted with an array of holes, and a second layer of thin metal, which is electron beam evaporated onto the patterned polymer to simultaneously create an array of disks and its complementary hole array. When excited at resonance, the coherent interaction of photons and the free electron density in the metal produce collective charge oscillations—termed surface plasmons. This interaction produces high-density charge localization on the edges of the metallic elements and microcurrents, whose energy dissipates by ohmic loss. The resonant wavelengths at which this occurs depends not only on the bulk metal dielectric function but also the polarizability of each metallic element, a lattice sum that characterizes the effect of near-field coupling, and the phase feedback from the reflective backplane when coupled with the Fabry-Perot cavity. Specifically, we define the structural parameter set as the pattern periodicity (*P*), the hole/disk diameter (*D*), the relief depth of each hole (RD), and the thickness of the cavity (L). A false color scanning electron microscopy (SEM) cross-section of the cavity-coupled plasmonic system, which depicts the various parameters, is shown in Fig. 1b. Images can be encoded in the surface within a desired spectral range while not appearing within others by properly varying at least one of the system’s parameters. Figure 1c illustrates this concept with a system designed for the mid wave infrared (MWIR) window. While a grayscale image appears when viewed through a MWIR camera, the appearance within the visible and long wave infrared (LWIR) regimes remain constant and uniform.

### Multispectral response

**Fig. 2:**
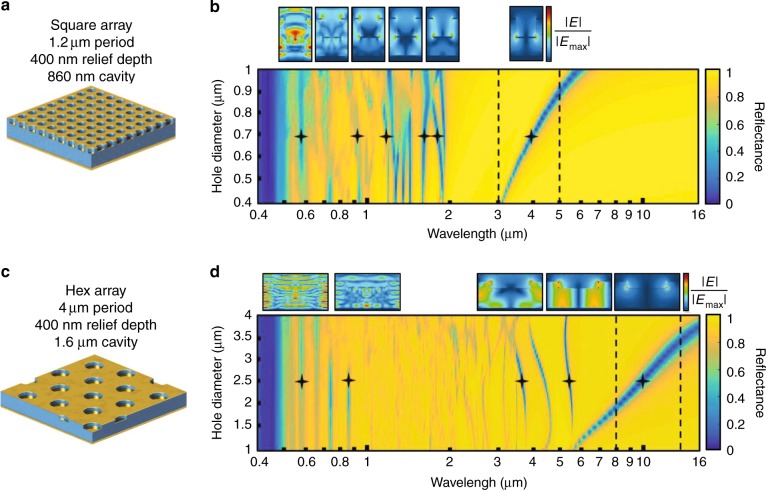
Parameter space exploration. Two systems are explored for operation in the mid wave infrared (MWIR) and long wave infrared (LWIR) transparency windows. **a** Schematic of the plasmonic device designed for the MWIR and **b** corresponding finite-difference time-domain (FDTD) simulations of reflectance as a function of hole diameter. **c** A schematic of the LWIR device and **d** the equivalent FDTD sweep of hole diameters. Dotted black lines depict the infrared desired range of operation. Hole diameter can be used to sweep through these windows while keeping visible absorption invariant. Field profiles are presented at labeled wavelengths and hole diameters to illustrate the mechanisms behind the resonances in different spectral regimes

The optical characteristics of the cavity-coupled plasmonic system can be categorized based on its geometrical features relative to the wavelength of incident light *λ*_inc_. To demonstrate this effect and the impact of the parameter set on the system’s electromagnetic response, we define and simulate two devices to operate in the MWIR (3–5 μm) and LWIR (8–12 μm) atmospheric transparency windows. Figure 2a, c shows schematics of these systems and their corresponding structural dimensions. The multispectral reflectance spectra of the respective surfaces as a function of hole diameter were calculated using the FDTD method, as shown in Fig. 2b, d. Vertical dotted black lines denote the desired infrared windows of operation. Field profiles of the systems are shown above the spectra for select resonant wavelengths and hole diameters. When *λ*_inc_ is considerably larger than the pattern lateral dimensions (*λ*_inc_ ≫ *D*, *P*), the system behaves as a metallic plane or mirror. As *λ*_inc_ decreases, extraordinary light transmission occurs through the subwavelength hole-disk array due to an induced plasmon resonance, which couples the electromagnetic wave into the cavity. The right-most field profiles above Fig. 2b, d depict this resonance, which occurs when the antinode, or constructive interference of the optical cavity mode, coincides with the location of the disk within the cavity and selectively polarizes the metallic disk. This feedback enhances the plasmonic fields on the edge of the disk, which eventually dissipates as electron resistive loss. The resonance and corresponding light absorption can be understood as a quasi-3D array of coupled dipole oscillators, which are directly influenced by all structural parameters and accurately modeled by the coupled dipole approximation (CDA)^16^.

As *λ*_inc_ becomes comparable to the array’s structural dimensions, the system supports higher-order plasmonic and interference resonances due to the onset of cavity-internal diffraction. For our devices, this finding corresponds to the NIR and visible regimes, *λ*_inc_ < *P*, where light diffracts into the cavity and couples to Fabry-Perot-induced plasmon modes supported on the disks and top metal film, which produces sharp and narrow dips in reflection^18^. These modes are primarily invariant to the hole diameter and relief depth while being highly sensitive to periodicity and cavity thickness due to the diffractive nature in which they are generated. The progression of modes from localized plasmonic oscillations to cavity- and diffraction-induced resonances is observed in the field profiles that accompany the spectra. Supplementary Fig. S1 and Supplementary Fig. S2 show the effects of relief depth and cavity thickness for the MWIR structure and LWIR structure, respectively. From this parameter study, the hole diameter and relief depth provide two possible routes for achieving infrared encoding while maintaining a uniform visible absorption.

### Role of cavity-external diffraction

**Fig. 3:**
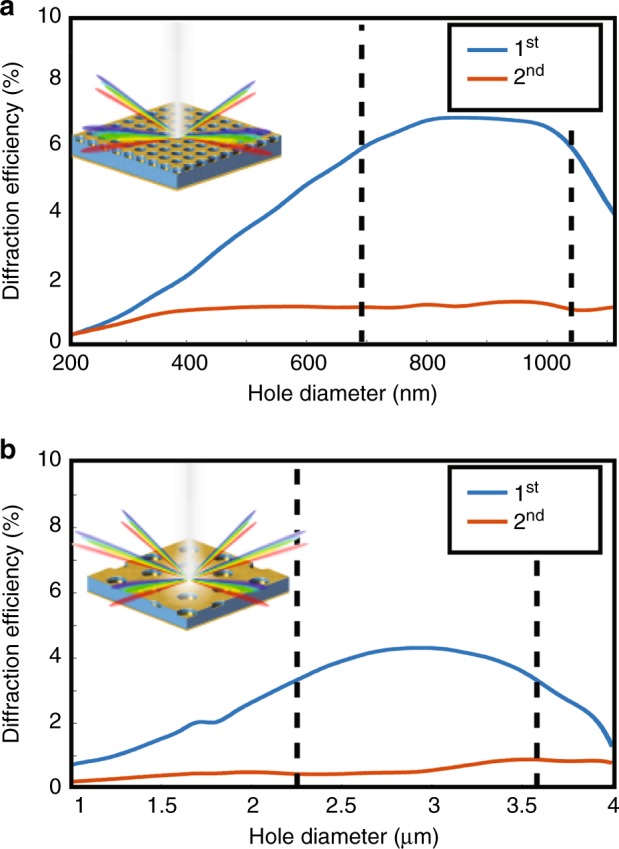
Pixel-to-pixel variation in diffraction. The spectral and order averaged diffraction efficiency of the first and second orders at normal incidence for **a** the MWIR device and **b** LWIR device. Efficiency is averaged over the visible spectral range of 400–800 nm. Black dotted lines indicate the range of diameters with a maximum deviation of 1%. Insets depict the respective devices and the degeneracy of the first diffracted order based on the symmetry of the hole array

Given the periodicity of the pattern arrays, diffraction from the top perforated-gold surface into free space has an important role in the visible perception of the surfaces. To conceal information encoded in the surface, diffracted light must not differ among pixels. This condition places on pixels the design constraint that periodicity be held constant, which ensures that the surface will have a uniform spectral distribution or color for any angle of incident light and viewing angle. However, the efficiencies of all diffracted orders are not guaranteed to be uniform across a given parameter space, which may produce a surface with uniform color but variations in brightness that correspond to the encoded information. To quantify this effect, we calculate the diffraction efficiency of the MWIR and LWIR devices as a function of hole diameter. Performed through FDTD, Fig. 3a, b shows the spectrally (400–800 nm) and intraorder averaged diffraction efficiency as a function of hole diameter, for the MWIR device and LWIR device, respectively. According to these results, the diffraction is dominated by the first-order modes, which have an efficiency range of 1–7% for the MWIR device and an efficiency range of 1–4% for the LWIR device in the visible spectral range. Note that this efficiency is the averaged strength of a single diffracted order and not their summation, because we aim to quantify the difference in light perceived by a single viewer at a given position. These results predict a near-invariant diffraction efficiency over the range of hole diameters Δ*D*, which is centered about the peak in efficiency. Black dotted lines indicate the range of hole diameters, in which the diffraction efficiency deviates by 1% or less. This range can be employed to calculate the span of wavelengths that can be traversed by the first-order resonance Δ*λ*. For the MWIR device, the analysis yields a Δ*D* of 350nm (*D* = 690–1040nm), corresponding to an infrared tunability Δ*λ* = 2.8 μm (*λ* = 3.9–6.7 μm). The same analysis for the LWIR device yields a Δ*D* of 1.33 μm (*D* = 2.25–3.59 μm) and LWIR tunability Δλ = 6.88 μm (λ = 8.96–15.84 μm). These Δλ values show that the structures can be primarily tuned throughout the infrared transparency windows by modifying the hole/disk diameter while minimizing pixel-to-pixel brightness variations in the visible domain.

### Device fabrication and characterization

**Fig. 4:**
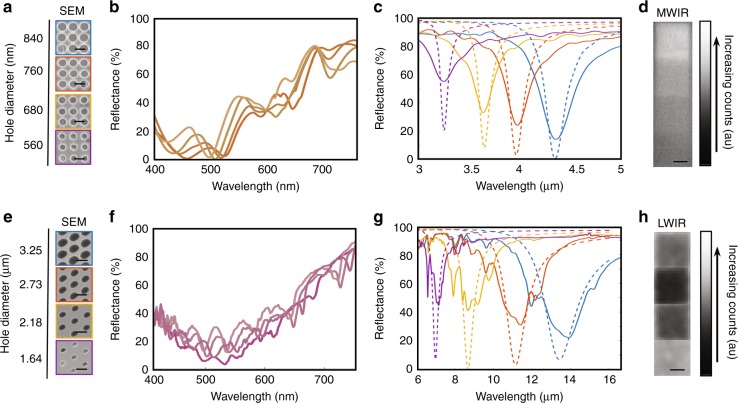
Experimental hole diameter sweep. Top-view scanning electron microscope images of the hole diameters of the fabricated plasmonic systems, visible and infrared spectrometer measurements, and infrared images of the **a**–**d** MWIR and **e**–**h** LWIR devices. Solid lines for the reflectance spectra are measured values, whereas dotted lines are obtained by FDTD simulations. Line colors for the visible spectra are obtained from the CIE chromaticity matching functions

To experimentally demonstrate cavity-coupled plasmonic devices, we utilize a combination of direct laser writing and nanoimprint lithography (NIL). While useful in rapid prototyping and scaling of the devices, the process involves several intricacies, which impact the accessibility of certain parameter spaces and the quality of resonances formed within them. Our method, however, constrains a device to a single cavity thickness and relief depth, leaving the hole diameter as the prime parameter to achieve multispectral encoding. Once the master is fabricated by direct laser writing into a spun-coated polymer, a cast is made using a soft polymeric material that is then employed to replicate the features of the master in spun coat films of thermoplastic (refer to the Materials and methods section for details). Electron beam evaporations of gold before and after the NIL process produce three-layered metallic cavities. Scanning electron microscopy images of the completed surfaces are shown in Fig. 4a, e for the MWIR device and LWIR device, respectively. By varying laser writing parameters, such as laser power and speed in the master writing process, a range of hole diameters are obtained in the end devices. The visible spectra of the surfaces are measured via a fiber-coupled spectrometer (Ocean Optics, HR 2000+) and a ×10 objective at normal incidence. Shown in Fig. 4b, f with line colors obtained by the CIE chromaticity matching functions, the variation in the hole diameter produces a small but measurable effect on the color of the surface in the visible domain. We attribute this effect to differences in cavity thickness, which are created by the nonequal SU-8 volume displacement of various hole diameters during the imprinting process. This effect can be mitigated by creating local height offsets within the stamp that is dependent on the hole diameter. The infrared reflectance spectra of the surfaces are obtained by a microscope-coupled Fourier transform infrared spectrometer (FTIR) and are represented by solid lines in Fig. 4c, g. Dotted lines indicate the spectra obtained by FDTD simulations. From these data, we observe the predicted redshift of the resonance with an increase in the hole diameter and a close match between the experimental resonance location and the simulation resonance location. However, the spectra slightly differ in the resonance strength and width. We attribute this difference to the ×15 Cassegrain objective that is used in experimental measurements. With a numerical aperture of 0.4, the angle of incident and collected light extends to ~41°—which causes an averaging effect of the resulting spectra over angle. This contrasts with the completely normal incident plane waves of the FDTD simulations. To demonstrate the system’s angle dependence and confirm the impact of wide angle objectives, we provide Supplementary Fig. S3, where we perform FDTD simulations as a function of angle and average the resulting spectra over 40° for unpolarized infrared light. In addition to angle induced broadening, distortions of resonance shape for the LWIR device is attributed to the intrinsic infrared absorption bands of SU-8^19–21^, where it was assumed to be perfectly dielectric for simulations.

The infrared resonant surfaces can be imaged with cameras designed for their respective bands of operation—a cooled indium antimonide detector (A8300sc, FLIR) for the MWIR surface in Fig. 4d and an uncooled VO_x_ microbolometer camera (HD-1024, St. Johns Optical Systems) for the LWIR surface in Fig. 4h. The infrared cameras produce images based on the power incident upon them and can be split into the light emitted by and reflected off of the imaged object/s^22,23^.

1 $${P = \mathop {\int }\limits_{\lambda _1}^{\lambda _2} B\left( {\lambda ,T_{\rm{Sample}}} \right)\varepsilon \left( \lambda \right)S\left( \lambda \right)d\lambda + \mathop {\int }\limits_{\lambda _1}^{\lambda _2} B\left( {\lambda ,T_{\rm {External}}} \right)R\left( \lambda \right)S\left( \lambda \right){\rm{d}}\lambda}$$

Both of these terms are given by an overlap integral of the surface’s emissivity/reflectivity ε(*λ*) and *R*(*λ*), the detector’s sensitivity spectrum *S*(*λ*), the blackbody spectrum at the object temperature and any possible external sources that reflect off the sample B(*λ,T*_Sample_) and *B*(*λ,T*_External_). To resolve the submillimeter patches of patterned surface, the samples are imaged with inverted telephoto lenses and the samples perpendicular to the detector and at room temperature. This sets the external blackbody source *B*(*λ,T*_External_) to approximately the blackbody emission of the detector. In the cooled MWIR detector image of Fig. 4d, *T*_Sample_ > *T*_External_, and the image is dominated by the emission of the sample. Kirchoff’s Law states that the thermal emission of an object ε(*λ*) is equivalent to absorption while in thermal equilibrium. For the completely non-transmissive samples, this result can be obtained by taking 1 − *R*(*λ*). Therefore, an increase in the absorption of the surface increases the overlap integral of ε(*λ*) and the spectral sensitivity of the packaged camera system, which produces a brighter or “hotter” image. The opposite situation is true for the uncooled 8–12µm detector image of Fig. 4h. The temperature of the detector is slightly higher than that of the sample, *T*_Sample_ > *T*_External_; thus, the image is dominated by the reflectance of the sample where the detector acts as its source. This domination indicates that an increase in the absorption of the plasmonic surface produces a darker or “colder” spot in the resulting image. However, if the absorption band moves beyond the sensitivity band of the camera, the overlap integral will decrease, and an inflection point will be observed. This condition is observed in the case of the LWIR sample, as shown in Fig. 4g, h, where the hole diameter and absorption continually increase with an increase in the hole diameter, but the brightness of the infrared image decreases and then increases. Due to the inverted lens configuration that is used to obtain a magnified image, the factory black-body-equivalent-temperature calibration is not valid, and we report in arbitrary counts.

### Visibly concealed infrared images

**Fig. 5:**
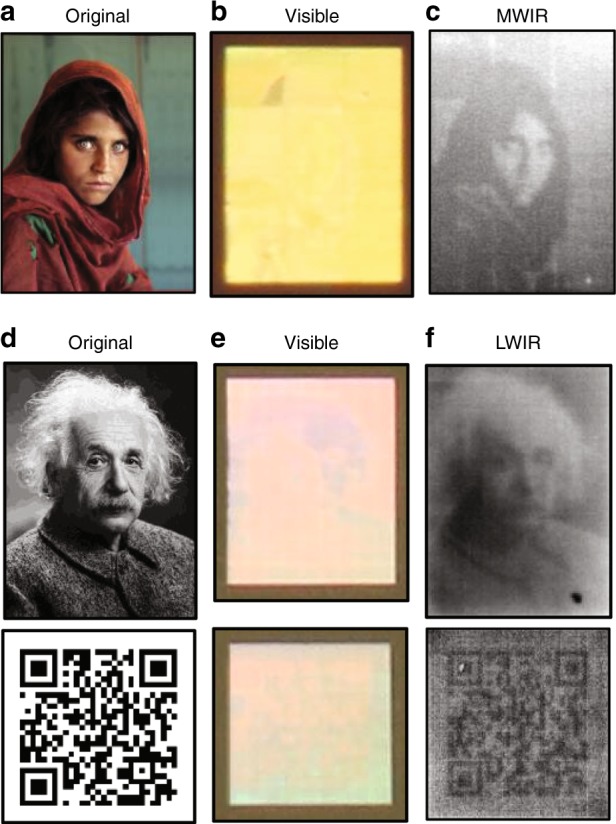
Infrared encoded data and images. **a** Image of the Afghan Girl (Copyright Steve McCurry/Magnum Photos. Image rights granted by Magnum Photos New York) which is encoded into the plasmonic surface by mapping hole diameter to infrared grayscale. **b** Visible camera (EOS Rebel T6i, Canon) and **c** infrared image of the encoded MWIR surface taken with a cooled indium antimonide detector (A8300sc, FLIR). The MWIR Afghan Girl encode device is 1 × 0.75 mm^2^ in size. The same procedure is conducted for the LWIR domain and images to be encoded are **d** that of Einstein and a QR code from our website (http:/nanoscience.ucf.edu/chanda). **e** A visible camera (EOS Rebel T6i, Canon) and **f** LWIR infrared images taken with an uncooled VOx microbolometer camera (HD-1024, St. Johns Optical Systems). The Einstein encoded sample area is 1.25 × 1 mm^2^, and the QR code is 1 × 1 mm^2^

To demonstrate the potential of the cavity-coupled plasmonic system, we encode images and data into the surface and observe through infrared and visible cameras. Fig. 5a depicts the well-known image of the Afghan Girl (Magnum Photos). Using the results of Fig. 4, we create a mapping between the hole diameters of a given pixel and the grayscale values of the surface when imaged through the infrared cameras. By direct laser writing, the image is encoded into the master and used in the sequential NIL process. The results in Fig. 5b, c are depicted as imaged through a camera (EOS Rebel T6i, Canon) and cooled InSb detector (A8300sc, FLIR), respectively. When viewed by the eye or a visible regime camera, the encoded surface appears as a uniform block of color that is dependent on the angle of incident light and viewing angle. However, the infrared camera depicts the intended grayscale image of the Afghan Girl. The resolution of the image is dependent on both the minimum pixel size of the plasmonic surface and the detector pitch of the infrared camera. The cavity-coupled plasmonic system requires multiple periodicities to effectively resonate and we have previously shown that three or more periods strongly absorbs^16^. We also provide Supplementary Fig. S4, which demonstrates the effect of pixel size on plasmonic resonance. We find an asymptotically increasing resonance strength and redshifting to that of the infinitely periodic case. For the MWIR sample in Fig. 5b, c, a pixel size of 10 μm × 10 μm, which equates to a 9-by-9 array of holes and disks is utilized. The detector pitch of the cooled InSb detector is 14 μm, which renders it and the lens assembly the limiting factors in potential images.

This procedure can also be completed for the 8–12 µm window device, where we arbitrarily choose an image of Einstein and a QR code as the data to encode, as shown in Fig. 5d–f show the surface viewed through a visible camera (EOS Rebel T6i, Canon) and an uncooled VOx microbolometer detector (HD-1024, St. Johns Optical Systems), respectively. Like the 3–5 µm band device, the hole diameter encoded surface appears as a near uniform field of color in the visible domain and appears as a grayscale image in the LWIR. A pixel size of 12 μm × 12 μm, which equates to a 3 × 4 array of holes and disks, is employed. The limiting factor in image resolution is the pitch of the detector—17 μm for the LWIR camera. Slight variations or outlines may appear in the visible images, as shown in Fig. 5e, due to changes in the diffraction efficiency among pixels of different hole diameters. This finding is more apparent in images with large contrasting regions or those with sharp transitions in the hole diameter. To minimize this effect, a smaller range of hole diameters can be employed, and their distribution should be chosen to ensure that resonances directly span the sharpest edge of the camera’s spectral sensitivity. This approach guarantees the largest change in detected power (image contrast) given a change in the hole diameter. Because the sensitivity of the camera and its noise equivalent temperature are system-level attributes that depend on lens assemblies, filters, ROIC settings, and the temperature of the sample and detector, we do not provide a universal value of image contrast or temperature per unit hole diameter change. We emphasize that the encoded information or image is not “invisible”. The dimensions of the plasmonic hole-disk system exceed the diffraction limit of visible light, and individual features are visible with high magnification objectives.

## Discussion

A multispectral plasmonic system in which infrared resonances can be independently tuned while keeping visible properties invariant has been demonstrated. This result is achieved via multispectral coupling mechanisms and the constraint of key structural parameters. The dipolar coupling between the array of holes/disks and their interaction with the optical cavity dictate the infrared response, whereas diffraction into Fabry-Perot cavity modes dominates the visible regime. We encode grayscale images and data into the surface by mapping the hole diameter of the plasmonic system to respective pixels. Combined with the ease of fabrication and compatibility on flexible substrates, this study can lead to new plasmonic surfaces with multispectral functionalities, such as information encoding for thermal management, camouflage and anti-counterfeiting.

## Materials and methods

### Fabrication of cavity-coupled plasmonic devices

The nanostructured surface is fabricated by nanoimprint lithography (NIL) for rapid replication. A polymer (dimethylsiloxane) (PDMS; Dow Corning, Sylgard) stamp is cast from a master, which has been constructed by direct laser writing. To control the cavity thickness of the samples, a thin film of SU-8 2002 (MicroChem) was spun for 1 min at 3000 rpm and then prebaked at 95 °C for 1 min. This film is imprinted with the PDMS stamp (20 s) on the hotplate. The stamp and substrate are removed from the hotplate and allowed to cool (30 s). After stamp delamination, the substrate is UV cured (1 min) and post-exposure baked (95 °C for 1 min).

### Direct laser writing of NIL master

Masters for the NIL process are created by direct laser writing using a Nanoscribe GT (Nanoscribe, GmbH). A glass slide is spun with a diluted mixture of Shipley S1813 (Shipley) and propylene glycol monomethyl ether acetate (PGMEA, Sigma Aldrich) (2:1) to create a 400nm film. The sample is pre-exposure baked at 100 °C for 2 min. DLW is performed with a ×20 air objective, and the sample is post-exposure baked for 3 min at 100 °C. The master is then developed in MIF 726 for 1 min. Variations in hole diameter are achieved by changing the intensity of the exposing beam. The large voxel of the air objective produces near vertical sidewalls, which are needed for a strong first-order plasmonic resonance because slanted sidewalls can create connections between the disks and the top perforated film.

### Electron beam deposition

The 150-nm-thick backmirror and 30-nm-thick top Au films are deposited using a Thermionics electron beam evaporation system. A 5-nm layer of Cr is evaporated for the backmirror to aid in adhesion to the glass substrates. Evaporations are performed at room temperature and at pressures of ~6×10^–6^ T with deposition rates of ~1 nm s^−1^.

### Infrared measurements and images

Reflection spectra are collected using a ×15, 0.4 numerical aperture Cassegrain objective on an optical microscope (Hyperion 1000) coupled to a Fourier transform infrared spectrometer (Vertex 80). Reflection spectra are normalized to a gold mirror. A cooled indium antimonide detector (A8300sc, FLIR) is used for the MWIR devices, whereas an uncooled VOx microbolometer camera (HD-1024, St. Johns Optical Systems) is used for the LWIR devices.

### Visible images

Photographs were taken using an EOS Rebel T6i Canon camera with an 18–55 mm lens and an additional ×10 macro lens. A sample was placed at ~5 cm from the camera lens with ~10º inclination. A tungsten bulb was employed as a background illumination source.

### FDTD modeling

Reflection spectra are calculated using the commercial FDTD software package (Lumerical FDTD, Lumerical Solutions Inc.). A plasma/Drude model is used for the dispersion of gold with DC permittivity of 9.5, a plasma resonance of 1.182 × 10^16^ (rad s^−1^) and a plasma collision frequency of 9.5 × 10^13^ (rad s^−1^). The results of these simulations are compared with those obtained from measured dispersions of CRC. A diffraction analysis is also obtained from FDTD.

## Electronic supplementary material

Supplementary Information

## Data Availability

The data that support the findings of this study are available from the corresponding author upon reasonable request.
