# Supplementary material for: Covert infrared image encoding through imprinted plasmonic cavities
Source: Light Sci Appl. 2018 Nov 21;7:93. doi: 10.1038/s41377-018-0095-9 (PMC6249251; doi:10.1038/s41377-018-0095-9)
Supplement: Supplementary file 1 — Supplementary Information [file 41377_2018_95_MOESM1_ESM.docx]

**Supplementary Information for:**

**Covert Infrared Image Encoding through Imprinted Plasmonic Cavities**

Daniel Franklin^1,2^, Sushrut Modak^2,3^, Alireza Safaei^1,2^, Abraham Vázquez-Guardado^2,3^, Debashis Chanda^1, 2, 3^


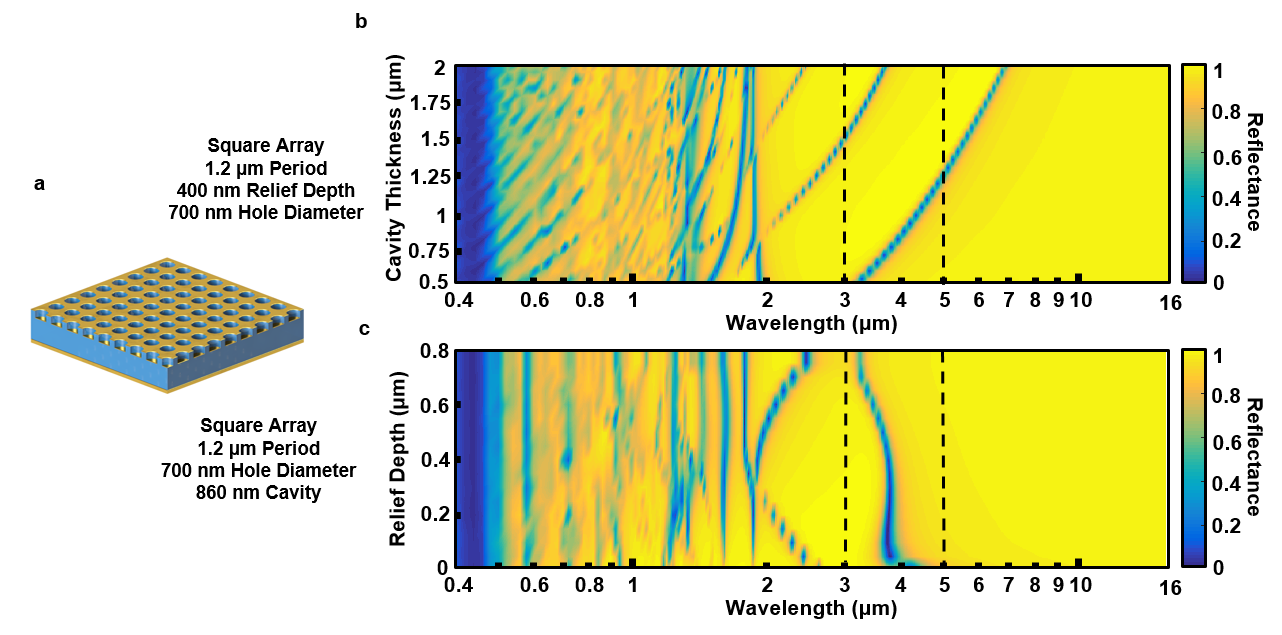


**Supplementary Figure 1 | Square array parameter space exploration**. (**a**) The three-layer plasmonic system designed for the mid wave infrared (MWIR) is systematically explored. (**b**) Finite difference time domain (FDTD) simulations of reflectance as a function of cavity thickness. (**c**) FDTD simulations as a function of relief depth. Dotted black lines depict the infrared desired range of operation.


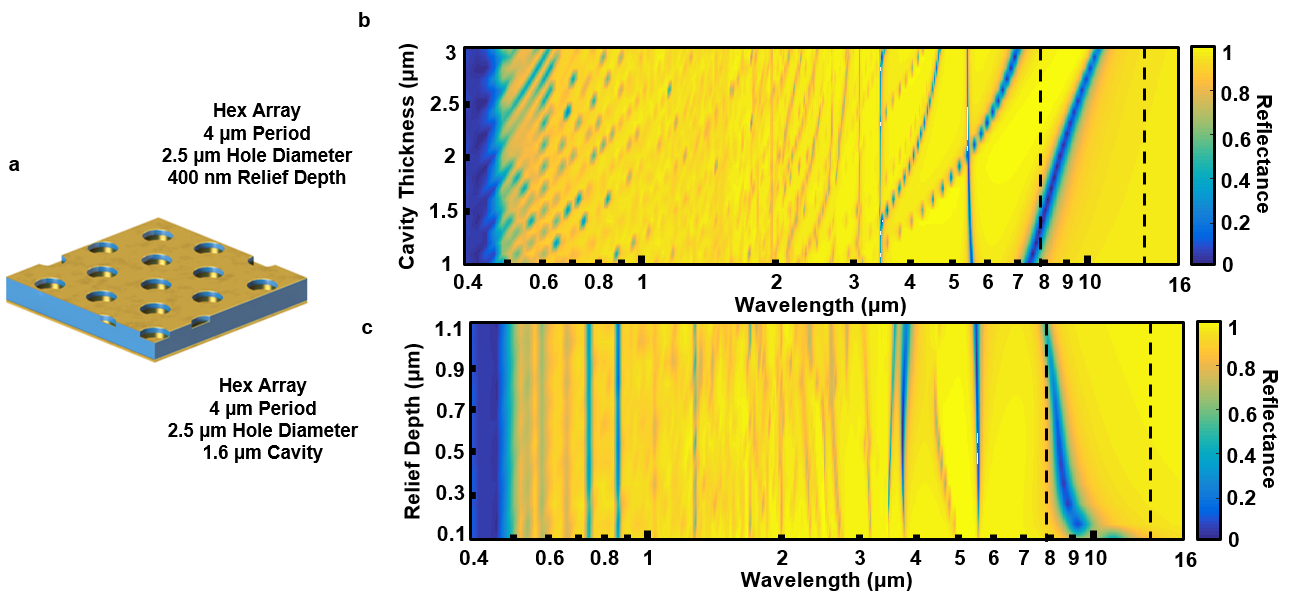
**Supplementary Figure 2 | Hexagonal array parameter space exploration.** (**a**) The three-layer plasmonic system designed for the long wave infrared (LWIR) is systematically explored. (**b**) Finite difference time domain (FDTD) simulations of reflectance as a function of cavity thickness. (**c**) FDTD simulations as a function of relief depth. Dotted black lines depict the infrared desired range of operation.


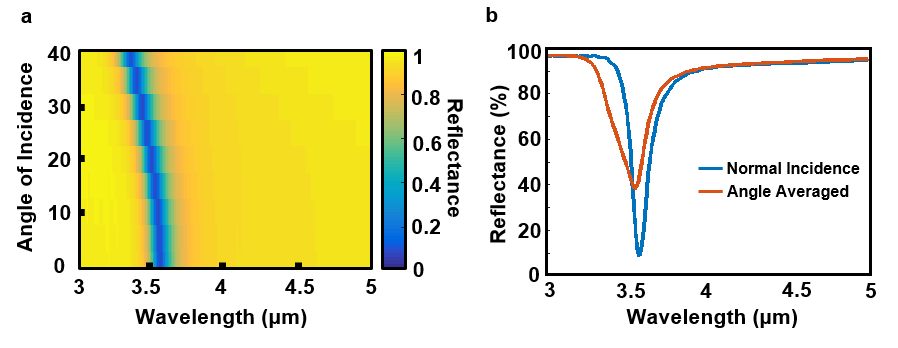


**Supplementary Figure 3 | Cavity Coupled Plasmonic Angle Dependence.** (a) Finite difference time domain (FDTD) simulations of the MWIR device as a function of incident angle. S and P polarization states are averaged and show a blue-shift as angle increases. (b) Simulated reflectance spectra of the surface for normal incidence and when angle-averaged over the 40o span – the angle attenuated by the 0.4 NA cassegrain objective used in experimental measurements. This accounts for decreased resonance amplitude and FWHM broadening observed experimentally.


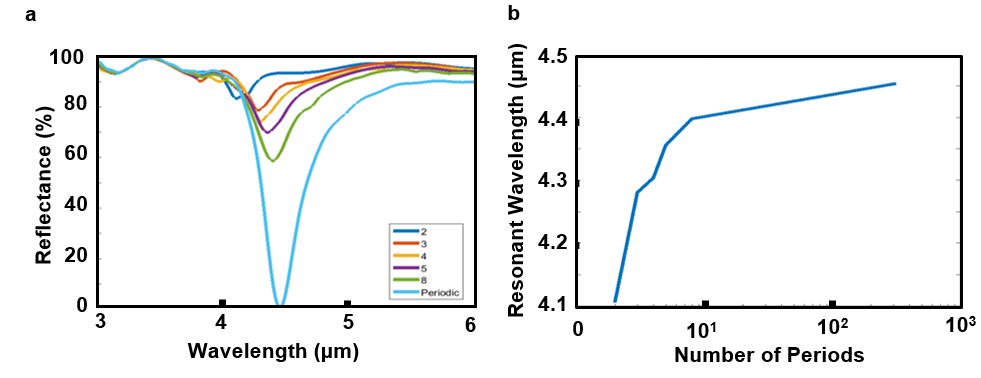


**Supplementary Figure 4 | Pixel Size Dependence.** (a) Finite difference time domain (FDTD) simulations of the MWIR device as a function of number of periods. Simulations are performed through the super cell method and by utilizing periodic boundary conditions but maintaining a 5 µm perimeter. These spectra are compared to the perfectly periodic case. The constant perimeter in simulations result in a changing cross section of the surface’s active region. Therefore, we track the resonant wavelength in (b) and see how the resonance is modified through physical truncation. While even a 2 x 2 is enough to produce a resonance, at least a 5 x 5 array is needed for the resonance to be within 100 nm of that for the infinitely periodic case.
